# A Review of Machine Learning Methods Recently Applied to FTIR Spectroscopy Data for the Analysis of Human Blood Cells

**DOI:** 10.3390/mi14061145

**Published:** 2023-05-29

**Authors:** Ahmed Fadlelmoula, Susana O. Catarino, Graça Minas, Vítor Carvalho

**Affiliations:** 1 Center for Microelectromechanical Systems (CMEMS-UMinho), Campus de Azurém, University of Minho, 4800-058 Guimarães, Portugal; id9247@alunos.uminho.pt (A.F.); scatarino@dei.uminho.pt (S.O.C.); gminas@dei.uminho.pt (G.M.); 2 LABBELS—Associate Laboratory, 4800-058 Guimarães, Portugal; 3 2Ai, School of Technology, IPCA, 4750-810 Barcelos, Portugal; 4 Algoritmi Research Center/LASI, University of Minho, 4800-058 Guimarães, Portugal

**Keywords:** FTIR spectroscopy, human blood cells, machine learning, review

## Abstract

Machine learning (ML) is a broad term encompassing several methods that allow us to learn from data. These methods may permit large real-world databases to be more rapidly translated to applications to inform patient–provider decision-making. This paper presents a review of articles that discuss the use of Fourier transform infrared (FTIR) spectroscopy and ML for human blood analysis between the years 2019–2023. The literature review was conducted to identify published research of employed ML linked with FTIR for distinction between pathological and healthy human blood cells. The articles’ search strategy was implemented and studies meeting the eligibility criteria were evaluated. Relevant data related to the study design, statistical methods, and strengths and limitations were identified. A total of 39 publications in the last 5 years (2019–2023) were identified and evaluated for this review. Diverse methods, statistical packages, and approaches were used across the identified studies. The most common methods included support vector machine (SVM) and principal component analysis (PCA) approaches. Most studies applied internal validation and employed more than one algorithm, while only four studies applied one ML algorithm to the data. A wide variety of approaches, algorithms, statistical software, and validation strategies were employed in the application of ML methods. There is a need to ensure that multiple ML approaches are used, the model selection strategy is clearly defined, and both internal and external validation are necessary to be sure that the discrimination of human blood cells is being made with the highest efficient evidence.

## 1. Introduction

As society continues to evolve, the importance of healthcare as a crucial pillar becomes more evident with each passing day. Providing and improving healthcare services has become an essential target, as it plays a vital role in supporting other societal pillars. Automation and medical devices are an integral part of healthcare services, and their significance has increased with advancements in technology and communication. It is now a prominent time for the healthcare industry to revolutionize the way it delivers services to society, given the rising number of diseases and epidemics.

With the population increasing every second, the demand for laboratory testing has also surged, requiring more health experts to attend to patient analysis and reporting. Particularly, in the case of complex elements such as blood, there is an urgent need for fast and accurate technology to provide initial indications of a patient’s status [1,2].

An adult human body contains approximately five liters of blood, with blood cells comprising nearly 45% of the blood tissue volume. Blood cells are categorized into three types—red blood cells (RBCs), white blood cells (WBCs), and platelets. In particular, the WBCs include basophils, lymphocytes, neutrophils, monocytes, and eosinophils. RBCs serve as the primary means of transporting oxygen, WBCs play a crucial role in the immune system, fighting against diseases, and platelets aid in the coagulation process, promoting wound healing with scabs. Both physiological and pathological changes can affect the composition of blood, which is clinically a crucial factor to consider [3].

Blood tests have emerged as a direct means of detecting an individual’s health status or diagnosing illnesses. Complete blood cell (CBC) counting is a traditional blood test that involves identifying and counting basic blood cells to examine, monitor, and manage variations in blood. Although this technology has been used since the last century, it has become burdensome for the healthcare sector due to the time, power, and reagents required [4].

It is now time to explore new technologies for analyzing human blood. For instance, Fourier transform infrared (FTIR) spectroscopy is a powerful, low-cost, and fast analysis tool. It can determine the molecular structure of a substance by matching the specific frequency absorbed by the molecule through the transition of the vibration frequency of the bond or group [4]. It has been used for human blood analysis. However, the interpretation of the output FTIR data is, in most cases, performed by human intervention. To overcome this limitation, researchers have joined the use of FTIR spectroscopy analysis to machine learning (ML) for the distinction between pathological and healthy human blood cells.

Thus, this paper aims to perform a review of articles that discuss the use of FTIR spectroscopy and ML for human blood analysis. The resulting tool can benefit healthcare providers as it utilizes precise and specialized equipment. With the use of ML algorithms, the procedure can be automated, replacing the need for specialized equipment.

Artificial intelligence (AI) is a broad scientific endeavor that involves developing computational systems to simulate human intelligence and enable problem-solving. ML and deep learning (DL) are subfields of AI, with the study herein presented focusing on ML. It is essential to understand the concept of AI before delving into each of these subfields, including their differences and applications. AI provides a means of automating various tasks without human intervention, making it a sought-after solution in many industries, including homes [5].

ML involves the use of computational tools and methods designed for specific goals to solve problems. In situations where the data input and the desired result are known, but the method to get there is unknown, the ML model can forecast and provide a solution. Therefore, ML presents a different approach from the traditional programming methods where the parameters and approaches are known [6].

ML models undergo data preparation, training, and testing stages. Data preprocessing involves examining and modifying data to improve model comprehension by removing irrelevant information and changing the format. To forecast any value or outcome, the ML model must first be trained using a specified approach. The user responsible for training must provide a substantial amount of data, as well as the expected results for each data point, along with training parameters and settings. This way, the model can learn to understand and interpret the data, making accurate predictions about future outcomes [7,8].

This paper is organized into 5 sections. Section 2 presents the methods used in the review, and how the papers have been collected and selected. Section 3 presents the results obtained from the collected data, which are analyzed and discussed in Section 4. Finally, Section 5 displays the paper’s conclusions and suggestions for future work.

## 2. Method

**Figure 1:**
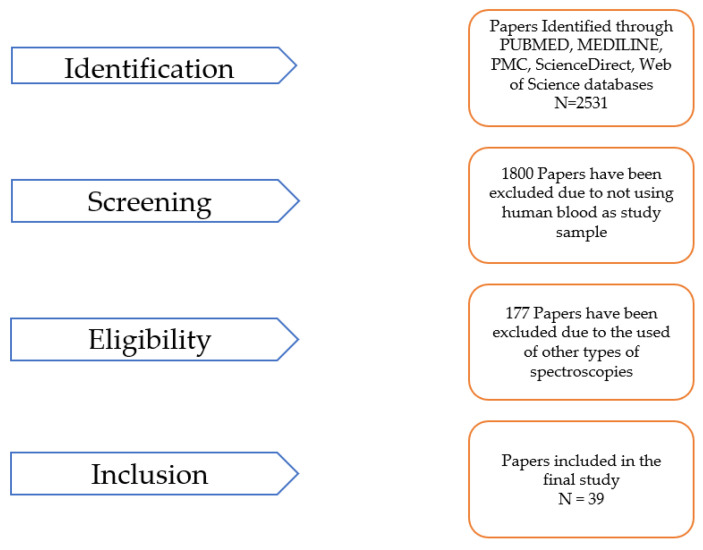
Schematization of the search and selection process. A total of 39 peer-reviewed articles from 2019 to 2023 were selected and reviewed.

For this paper, a review was conducted using the standard methodology illustrated in (Figure 1). The following databases were searched for peer-reviewed journal articles published between 2019 and 2023: PubMed, MEDLINE, PMC, ScienceDirect, and Web of Science. Before 2019, the number of papers representing ML methods applied to FTIR spectroscopy was low (the authors only identified four papers under this criterium) and, consequently, not significant [9,10,11,12], so this time period was not considered in the search process. After 2019, the number of papers increased dramatically, and the quality of the data showed promising outcomes [13,14], allowing the availability of data to perform a more detailed review analysis. In addition to the database search, relevant articles were manually identified by reviewing the reference lists of the included articles to investigate the effectiveness of the reference lists for the identification of additional, relevant studies. The search terms used were “Machine learning”, “Machine learning AND FTIR OR Attenuated Total Reflectance (ATR)-FTIR spectroscopy”, and “Machine learning AND FTIR OR ATR-FTIR spectroscopy AND human blood”. No language restrictions were applied, and Excel software was used to store the articles retrieved from the databases and screen for duplicates. Eligible studies included those that analyzed prospective or retrospective observational data, reported quantitative results for the experiment and evaluation made for the method used. The papers’ data were separated into two tables, whereby in one table (1), the study’s targeting disease, the study type, the sample source methodology, and the FTIR analysis, among other details, are described, and the ML methods, metrics, and validation approach are described in another table (2).

The information extracted from the articles was analyzed descriptively and qualitatively and grouped into categories such as study characteristics, diseases studied, statistical methodologies employed, software packages, as well as the strengths and weaknesses of the reported studies. The findings were interpreted based on these categories (Section 3. Results).

## 3. Results

**Figure 2:**
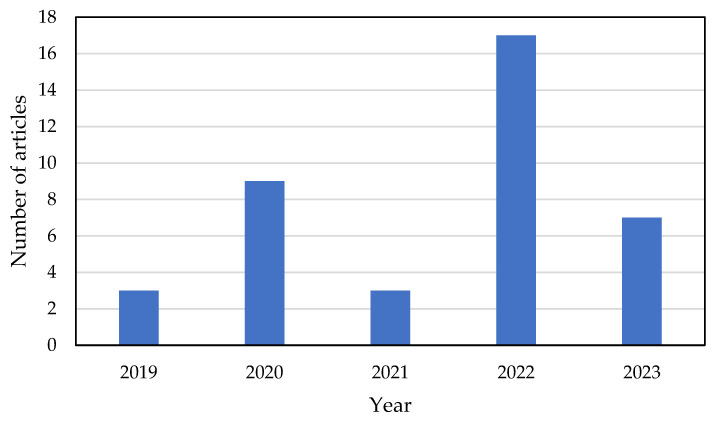
Counting of articles, in the last 5 years, relating to ML and FTIR spectroscopy applied to human blood cells.

The search methodology described in Section 2 was conducted to identify articles published in the last five years (2019–2023) that combined FTIR spectroscopy and ML methods to distinguish between human blood cells. A total of 39 eligible studies were identified and their characteristics, along with patient data, were summarized based on the keywords used in the search. Figure 2 illustrates that, in the last five years, there has been a growing interest among scientists and researchers in the medical field in the application of AI and ML for FTIR data analysis. These studies have demonstrated the potential of AI and ML to improve healthcare services, particularly in the area of disease diagnosis based on human blood.

### 3.1. Summary of the Eligible Publications

Table 1 summarizes, with a chronological criterium, the sum-up content data from the 39 selected papers. It includes information about the targeted diseases, the criteria followed in the data collection process, the methodology to collect the samples, and the sample size, as well as the used software and positive and negative outcomes from each reported study.

The presented data (see Figure 2) show that in 2022, the number of papers published on the application of ML and FTIR for diagnosing multiple diseases reached an all-time high. This was particularly evident after the pandemic in 2020, which acted as a catalyst for the adoption of ML and FTIR technologies. The majority of the data sources used in these papers were based on real experimental datasets or designs, accounting for 75% of the total, while the remaining 25% primarily utilized electronic health records (Table 1).

### 3.2. ML Methods, Metrics, and Internal Validation in the Selected Papers

Table 2 illustrates, also chronologically, the reported ML methods, metrics to evaluate ML, and internal validation procedures followed in the selected studies (according to the papers mentioned in Table 1).

Table 2 outlines the various ML algorithms used for classification or prediction in the selected studies, including SVM (featured in 24 studies), PCA (featured in 17 studies), the KNN and XGB [50] (featured in 6 and 5 studies, respectively), RF [51] (featured in 11 studies), PCA-LDA and OPLS-DA (featured in 5 and 4 studies, respectively), and LDA [52] (featured in 6 studies). The other algorithms/methods presented in the studies were only reported once.

**Figure 3:**
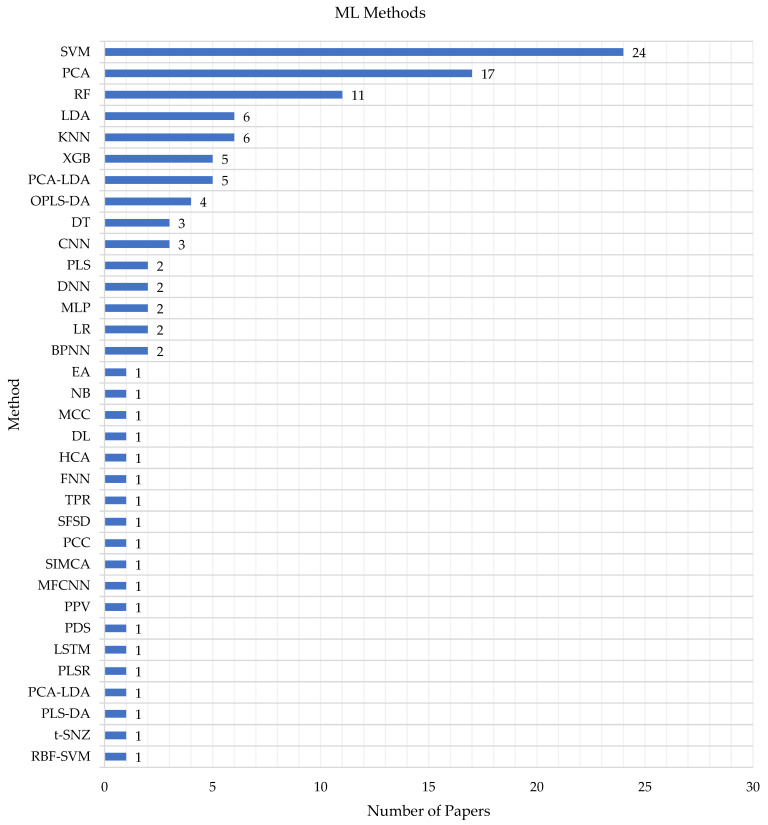
Frequency of the ML methods used in the eligible studies.

Figure 3 reveals that, in addition to the commonly used algorithms, other methods were employed, such as DT, BPNN, MLP, NB, LR [53], and a novel Bayesian approach, as well as various analytical approaches, including HCA, PCC, and PPV.

The figure shows that the SVMs are frequently used in the biological field—SVMs are one of the most powerful classifiers in ML that can be applied when a dataset is introduced in two classes in a high dimensional feature space, and this is the nature of biological cells.

Most biological genomic data are high-dimensional, heterogeneous, and noisy. This feature makes some methods such as SVMs, PCA, and RFs suitable to be used in the biological field rather than other such as the DL and PCC. 

In addition, as summarized in Table 2, almost all the publications (n = 33, 92%) utilized two or more methods, and only less than eight percent (n = 6, 8%) applied a single ML algorithm.

**Figure 4:**
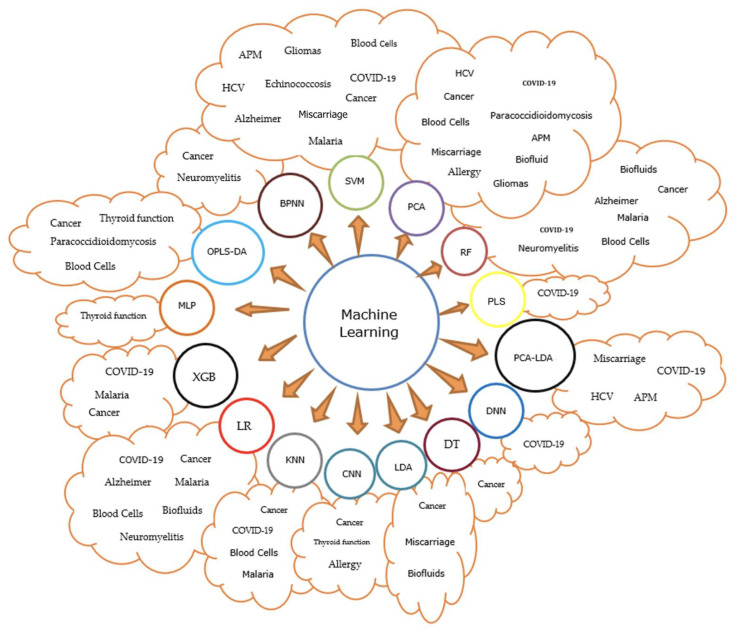
Graphical schematization of the most frequently used ML methods in the FTIR analysis of different diseases.

Additionally, further information is presented in Figure 4 to help establish a visual analysis between the type of diseased and the ML methods used for its classification.

### 3.3. Internal and External Validation 

The evaluation of the reported studies’ publication quality identified the most common gap in publications as the lack of external validation, which was conducted by only two studies [13,49]. Twelve of the reported studies predefined the success criteria for model performance [15,19,22,25,26,36,38,41,43,47,48] and nine studies discussed the generalizability of the model [14,17,20,27,29,33,35,42,46]. All the studies, except one [37], discussed the balance between model accuracy and model sensitivity and specificity. 

### 3.4. Strengths and Weaknesses 

The authors of the selected articles noted both strengths and weaknesses in the used ML methods. Overall, the simplicity and low complexity of ML methods were recognized as strengths, as they are powerful and efficient tools for handling large datasets. However, one article highlighted that the effectiveness of ML is highly dependent on proper method selection and parameter optimization and that these steps are essential for obtaining accurate estimates [25].

Even with careful planning and despite their advantages, ML approaches still present several limitations, which warrant attention in future studies. Overfitting was identified as a weakness, which can occur when too much detail is included in the method. Other limitations stem from the quality and availability of the data sources used, such as incomplete variable sets or missing data, which can negatively affect model development and performance. Retrospective database studies were identified as particularly vulnerable to the lack of relevant variables, as researchers are limited to recorded data. Finally, the lack of external validation was noted as a limitation of the studies reviewed in this analysis.

## 4. Discussion 

In this review, we examined the methods and approaches used for ML in the context of observational datasets related to the FTIR or ATR-FTIR analysis of human blood cell conditions. While ML methods have been applied more broadly in recent years, our review focused specifically on studies that utilized FTIR or ATR-FTIR spectroscopies and human blood cells, and therefore, our findings may not be applicable to all ML methods. Our primary objective was to explore the potential of ML methods in distinguishing between healthy and pathological cells on a large scale, not limited to a specific disease, to improve healthcare services and provide physicians with reliable evidence applicable to individual patients. This review aims to provide guidance and best practices for the use of ML methods in discriminating between human blood cells, with the goal of improving their effectiveness and increasing their use in generating data and models for healthcare decision-making. The used methods represent a single point on a potentially wide distribution, meaning that any cell spectrum could fall anywhere within that distribution and may be far from the point that distinguishes between healthy and pathological conditions.

Multiple algorithms were used in the majority of the articles, although in some articles single modeling methodologies were considered; this underscores the importance of selecting and developing ML algorithms, particularly considering recent advances in analytics capabilities. 

The FTIR analysis presented in Table 1 shows that all the studies discussed utilized the mid-IR domain, specifically the wavelength range of 400 to 4000 cm^−1^, with spectral resolutions ranging between 2–8 cm^−1^ being the most common 4 cm^−1^. While the basic IR parameters were consistent across the studies, the analysis varied in terms of the preprocessed spectrum region, i.e., the main interest regions. This variation is primarily influenced by the specific wavelength being targeted, which in turn affects the intensity band observed in the selected preprocessed spectrum region.

While a single model may sometimes produce accurate results that match the data well, creative methods can be used to support the model’s certainty. It is advised that this be adopted as a best practice in the future and be used as an extra criterion to evaluate the caliber of research among ML algorithms.

The methods that were used in each publication performed differently based on many inputs, varied in the metrics and internal validations, and gave different results describing the potential and significance of using these methods in the medical field.

SVMs and PCA are the two ML most frequently used methods in the biological field, with SVMs being used in 25 papers among the 39 selected ones and PCA being used in 17 papers. Other methods such as RFs, LDA, and XGB also play a big part in the biology field as methods for assessing biological cells and enhancing outcomes. 

Cancer seems to be the main targeted disease in order to develop a method that can be used to detect the carcinogenic cells on the nuclear stage (10 types of ML methods were used in the 39 selected papers), followed by inflammatory diseases in general. 

The classification average accuracy of identification/distinction between FTIR spectra was found to be 93.7% for cancer, 88.10% for COVID-19, 95.10% for Alzheimer’s disease, 93.90% for allergies, 93.30% for Aspergillus, 100% for miscarriage, neuromyelitis, hepatitis C virus, and Paracoccidioidomycosis, 95.3% for blood cells, 99% for echinococcosis, 91.62% for malignant pleural mesothelioma, 87.07% for gliomas, and 91.62% for biofluids (calculated from the data presented in Table 1).

**Figure 5:**
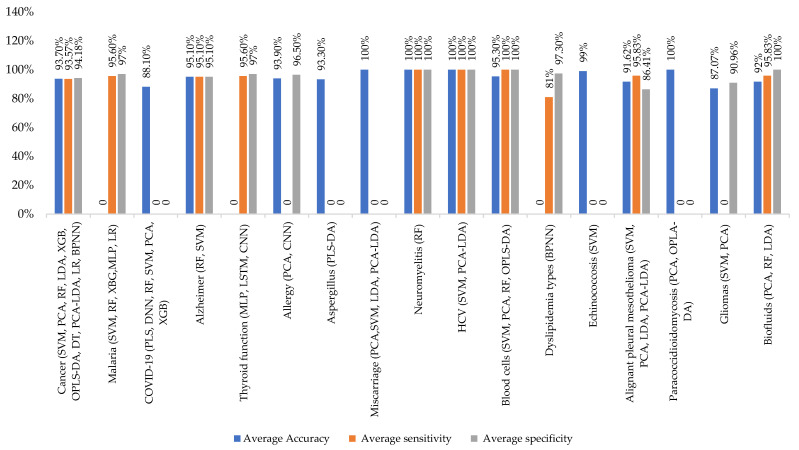
Metrics to evaluate the average ML performance in FTIR applied to different diseases. For each disease, the most common ML methods are reported. The blue column represents the average accuracy, the red column represents the average sensitivity, and the green column represents the average specificity.

These findings demonstrate the power of basic ML algorithms as applied intelligence tools to distinguish the complex vibrational spectra of, for example, cancer patients from those of healthy patients. These experimental methods show promise as a valid and efficient liquid biopsy for artificial-intelligence-assisted early cancer screening, as shown in Figure 5.

By combining FTIR spectroscopy with deep learning, it was possible not only to differentiate between allergic and healthy patients but also to stratify and treat patients, indicating its potential for monitoring the efficacy of SIT in individual patients. However, further investigation is needed to determine whether FTIR-spectroscopic-based identification of allergic status is limited to adults or can also be applied to samples collected from other groups, such as children. 

The classification process utilizing PLS and DNNs [54] algorithms shows promising results in distinguishing COVID-19 patients from healthy individuals. The extraction process revealed numerous features of the FTIR signal and combining all these features achieved effective accuracy values. 

The results indicate that using these 16 features could be valuable in accurately classifying COVID-19 patients and healthy subjects. For the proposed method for predicting the biological contour shape, the method enables the application of genomic selection to rice grain shape improvement as depicted in Figure 5.

Moreover, Figure 5 shows that the neural network models have successfully identified unique infrared absorption spectra in many diseases, such as cancer, allergies, thyroid function, and COVID-19. These spectra can effectively distinguish between malignant and benign breast tissues. 

The ML classification that used SVMs [55] to distinguish between pathological and healthy patients for multiple diseases such as malaria and Alzheimer’s disease and miscarriage achieved sensitivity between 95–100% (with three false negatives for each disease) and specificity between 95–97% (with two false positives for each disease). The method had good predictability with a low error rate and can be sufficiently accurate for the analysis of blood cells like the reference method. The suggested method is simple, fast, economical and does not require pollutant solvents and expensive equipment. It is worth noting that one of the false positives was due to a Plasmodium species other than Plasmodium falciparum or Plasmodium vivax, which was not detected by the PCR primers employed. Therefore, the results may actually be better than reported. The study also demonstrated that ATR-FTIR spectroscopy can be a reliable and efficient diagnostic tool for miscarriage, with the potential to be used at the point of care in tropical field conditions. The spectra can be analyzed via a cloud-based system, which makes it easily accessible for mass screening. This approach is highly sensitive, selective, portable, and requires low logistics, which makes it a potentially outstanding tool for malaria elimination programs. Currently, the experimental program is focused on reducing the sample requirements to fingerpick volumes [8].

## 5. Conclusions

This paper has presented a review of articles that discuss the use of FTIR spectroscopy combined with ML for human blood analysis between the years 2019–2023.

The reported implementation of ML techniques covered a wide range of approaches, methods, statistical software, and validation strategies. Based on these findings, it is essential to assess and compare several modeling approaches when creating ML-based models for correctly evaluating FTIR data, aimed at diagnosing human blood, which calls for the highest research standards. Models should be assessed using precise criteria before being chosen. 

There is potential to apply FTIR-based ML diagnosis to aid clinical decisions as a triage method for human blood cell discrimination, extending to early cancer screening, health monitoring, disease detection, and food safety. Identifying early stages or referral decisions is essential, usually unmet by the current diagnostic pathway. That level of proof has been attained by a sizable number of studies that distinguish between abnormal and healthy human blood cells. Moreover, considering data availability, power computing, and access to AI tools, it should be expected that there will be a high increase in the use of ML and DL approaches for human blood analysis in the future years due to the advantages they provide.

This fast method is highly important and critical for many patients. It is effective for both accessible and inaccessible diseases in which lab tests are normally useless. Thus, it could serve as a significant and objective diagnostic tool that will assist physicians in increasing their diagnostic accuracy of the etiology of diseases, especially for inaccessible patients. Before ML approaches are used to analyze blood cells, we need additional field validation in other study sites with different parasite populations and an in-depth evaluation of the biological basis of blood cells. 

ML approaches still present several limitations, which warrant attention in future studies using these methods. Overfitting has been identified as a weakness, which can occur when too much detail is included in the method. Other limitations stem from the quality and availability of the used data sources, such as incomplete variable sets or missing data, which can negatively affect the model’s development and performance. Retrospective database studies were identified as particularly vulnerable to the lack of relevant variables, as researchers are limited to recorded data. Moreover, the lack of external validation was noted as a limitation of the studies reviewed in this analysis. Thus, improving the classification algorithms and model training on larger datasets could also improve specificity and sensitivity, as well as looking up details to increase the potential of FTIR spectroscopy and ML application in the biological field [56,57,58,59]. Finally, the output from this paper—apart from the description of the most used ML techniques and corresponding metrics in the biological field—could be a strong basis for further developments, focusing the application of these methodologies on human blood cell analysis [60], even targeting their application in microfluidic, lab-on-a-chip [61,62,63,64], or other point-of-care miniaturized devices [65], among others. 

## Figures and Tables

**Table 1:** Summary of the eligible publications.

Reference	Targeting Disease	Study Type	Sample Source Methodology	Software	FTIR Analysis	Pros	Cons
Emmanuel P. Mwanga, 2019 [13]	Malaria	Cross-sectional study	1486 households and 3292 individuals	OPUS 8.7 SP2 software	Scanning range from 4000 to 500 cm^−1^ with the spectral resolution set at 5 cm^−1^ and the preprocessed spectrum region (main peaks identified in the 1730 to 883 cm^−1^ region). Analysis parameter: full spectra absorbance amplitude	Demonstrated that mid-infrared spectroscopy coupled with supervised ML could be used to screen for malaria parasites in human dried blood spots	Reduced the number of dataset samples for training which limits the prediction quality
Philip Heraud, 2019 [14]	Malaria	Pilot study	318 patients	MATLAB-based graphical user interface (GUI) (Release 2018b)	Scanning range from 4000 to 650 cm^−1^ at a spectral resolution of 4 cm^−1^ (main analysis in the 1800 to 800 cm^−1^ and 3200 to 2800 cm^−1^ regions). Analysis parameter: 255 combinations of the spectral peaks combined with 12 pre-processing methods (including first- and second-order derivatives and mean-centered, among others)	Showed potential as an efficient and reliable malaria diagnostic tool at point-of-care (POC) under tropical field conditions	Required more experimental work
Suat Toraman, 2019 [15]	Colon cancer	Clinical study	70 patients	MATLAB (Release 2018b)	Scanning range from 4000 to 450 cm^−1^ with 32 scans and 4 cm^−1^ resolution. Analysis parameters: area, height ratios, and six statistical features of the FTIR peaks	Achieved promising results for the classification process in distinguishing patients with colon cancer from healthy subjects	Weak understanding of the effect on the obtained signal of the water in the plasma
Adam H. Agbaria, 2020 [16]	Infection etiology in febrile pediatric oncology	Clinical study	116 patients	OPUS 7.0 software	Scanning range from 4000 to 600 cm^−1^ with 128 scans and 4 cm^−1^ spectral resolution (main peaks identified in the 1800 to 900 cm^−1^ and 3020 to 2880 cm^−1^ regions). Analysis parameters: normalized average spectra and second derivative of the spectra (using the Savitsky–Golay filter)	Infrared spectroscopy combined with ML algorithms was demonstrated as a powerful clinical tool for the diagnosis of the etiology of infection	Required more experimental work
Ahmad Salman, 2020 [17]	Lewy bodies and Alzheimer’s diseases	Clinical study	56 individuals	OPUS 7.0 software	Scanning range from 4000 to 600 cm^−1^ with a spectral resolution of 4 cm^−1^ (main peaks identified in the 1760 to 950 cm^−1^ region). Analysis parameters: normalized and baseline corrected average spectra and second derivative of the spectra	The combination of IR spectroscopy and ML allowed for differentiation between DLB and AD with a high rate of success	The classification results were based only on WBC samples
Zozan Guleken, 2020 [18]	Analysis of oxidative/antioxidative	Comparison study	30 individuals	Python Keras library version 2.3	Scanning range from 4000 to 600 cm^−1^ with an average of 32 scans and at a spectral resolution of 4 cm^−1^. Analysis parameter: ratios between the baseline-corrected and normalized spectra (using the Savitzky–Golay algorithm)	The spectroscopic method proved to be an effective tool to identify toxicological changes in the blood and serum of individuals with substance use disorder	Required the participation of a large number of patients from different clinical centers
Elke Korb, 2020 [19]	Allergy	Proof-of-concept study	10 individuals	GraphPad Prism 6, MATLAB (Release 2018b), and OPUS 7.8.5 software. Unscrambler X 10.5 software and Python programming language with Scikit-learn library	Scanning range from 4000 to 500 cm^−1^ using a 6 cm^−1^ spectral resolution (main peaks identified in the 1800 to 900 cm^−1^ region). Analysis parameters: absorbance amplitude values and second derivative spectra, calculated by the Savitzky–Golay algorithm	Allowed not only to differentiate between allergic and healthy patients but also stratified specific immunotherapy (SIT)-treated patients	Limited to adults
Adam H. Agbaria, 2020 [20]	White blood cells	Clinical study	343 individuals	OPUS 7.0 software	Scanning range in the mid-IR, from 4000 to 600 cm^−1^, with 128 scans at a 4 cm^−1^ spectral resolution. From each sample, 16 spectra from different sites were measured (main peaks identified in the 1800 to 900 cm^−1^ region). Analysis parameter: spectra derivative based on the Savitzky–Golay filter, applied on the normalized average spectrum	A significant and objective diagnostic tool that assisted physicians in increasing their diagnostic accuracy regarding the etiology of infections, especially for inaccessible infections	Needed more data to increase the diagnosis success rates
Feilong Yue, 2020 [7]	Thyroid function	Comparative study	199 patients	Not specified	Scanning range in the mid-IR, from 3000 to 700 cm^−1^, at a 4 cm^−1^ spectral resolution. Analysis parameter: amplitude of the infrared absorbance peaks	The deep learning model proved that it had the possibility for practical application	Required more experimental work
Hyunku Shin, 2020 [21]	Lung cancer	Clinical study	47 patients	Python version 2.3	Scanning range from 2000 to 475 cm^−1^. Analysis parameter: amplitude of the normalized Raman signals	Proved useful in identifying early-stage lung cancer patients	Used as a routine pre-screening tool for lung cancer
Zozan Guleken, 2020 [22]	Opioid use disorder	Clinical study	34 patients	Graph Pad Prism 6.01, Python software	Scanning range from 4000 to 450 cm^−1^ with 32 scans and at a 4 cm^−1^ spectral resolution (main peaks identified in the 1800 to 900 cm^−1^ region). Analysis parameter: spectra derivative based on the Savitzky–Golay filter, applied on the normalized average spectrum	FTIR for a simple and readily available diagnostic test that can successfully differentiate the serum samples of opioid use disorder patients from healthy subjects	The necessity of further studies on the effects of opioid use on the human body
Hugh J. Byrne, 2020 [23]	Bodily fluids	Multiple proof-of-concept studies	25 patients	MATLAB (Release 2017a)	Scanning region from 1800 to 400 cm^−1^ at a 4 cm^−1^ spectral resolution. Analysis parameter: amplitudes of the spectra derivative based on the Savitzky–Golay filter, applied on the normalized average spectrum	Demonstrated the potential for the clinical translation of quantitative serum analysis using vibrational spectroscopy	Increased clinical-scale trials for the in-depth assessment of the associated clinical workflow and health economics
Ashton G. Theakstone, 2021 [24]	Biofluids	Clinical study	Not reported	Python version 2.3	Scanning range from 3500 to 900 cm^−1^	FTIR spectroscopy techniques detected the early stages of disease, including before clinical symptoms arise, by analyzing samples at the molecular level	Required greater sample populations to validate the clinical potential of the proposed technologies.
Fangfang Chen, 2021 [25]	Gliomas	Clinical study	30 patients	MATLAB (Release 2016a)	Scanning range from 4000 to 500 cm^−1^ with 6 scans and at a 8 cm^−1^ spectral resolution (main peaks identified in the 3500 to 500 cm^−1^ region). Analysis parameter: average amplitudes of the normalized spectra	Mid-infrared spectroscopy with ML proved to be suitable for detecting cancer	Required more experimental work
Omar Anwar Elkadi, 2021 [26]	Aspergillus	Clinical study	45 samples	Unscrambler X 10.4	Scanning range from 4000 to 449 cm^−1^ at a 4 cm^−1^ spectral resolution. Analysis parameter: absorbance at each wave number	FTIR spectroscopy and ML identified with high sensitivity and specificity blood plasma with Aspergillus	Required more experimental work
Rock Christian Tomas, 2022 [27]	Breast cancer	Clinical study	200 samples	MATLAB (Release 2020b)	Scanning range of 4000 to 600 cm^−1^ with an average of 48 scans and at a 4 cm^−1^ spatial resolution (main peaks identified in the 1800 to 850 cm^−1^ region). Analysis parameter: absorbance amplitude of the normalized spectra	Neural network (NN) models can effectively discriminate malignant from benign breast tissues	Financial constraints
Abicumaran Uthamacumaran, 2022 [28]	Carcinogenic cells	Pilot study	9 patients	OriginPro 2021 and Scikit-learn Python library	Scanning range from 3040 to 1440 cm^−1^ at a 4 cm^−1^ spectral resolution. Analysis parameter: peak amplitude identification in the baseline corrected spectra (using the asymmetric least-squares smoothing method)	Accurately distinguished cancer EVs from those of healthy patients	Only investigated cancer EVs obtained from cancer cell lines
Zozan Guleken, 2022 [29]	COVID-19 antibody level	Clinical study	47 patients	OPUS 7.0 software	Scanning region from 4000 to 400 cm^−1^ with 32 scans and at a 4 cm^−1^ spectral resolution. Raman recorded in the 3700 to 150 cm^−1^ range with 64 scans and at an 8 cm^−1^ spectral resolution. Analysis parameter: absorbance amplitude (partial least-squares analysis), after normalization and smoothing with the Savitsky–Golay filter	Proved the possibility of using FTIR and Raman spectroscopy to identify differences in COVID-19 patients	Required more experimental work
O.K. Gasymov, 2022 [30]	Lung carcinoma	Comparison study	81 samples	Metabo Analyst 4.0 software	Scanning range from 4000 to 400 cm^−1^ with 512 scans and at a 4 cm^−1^ spectral resolution (main groups identified around 800 cm^−1^, 1100 cm^−1^, 1400 cm^−1^, and 3050 cm^−1^). Analysis parameter: area of the normalized spectra (bio-fingerprint and lipid) regions	Multivariate statistics with feature extraction and ML were successfully applied to cancer classification based on the FTIR spectra of blood plasma samples	Required a widespread screening method for the identification of individuals at high risk
Yu Du, 2022 [31]	Breast cancer	Clinical study	526 samples	KS algorithm OLS3	Scanning range from 4000 to 400 cm^−1^ with 32 scans and at an 8 cm^−1^ spectral resolution (main bands identified in the 1425 to 900 cm^−1^ and 1710 to 1475 cm^−1^ regions). Analysis parameter: peak intensity of the normalized spectra	FTIR and ML can not only accurately detect invasive breast cancer but also accurately distinguish ductal carcinoma in situ from invasive breast cancer and healthy controls	The authors need to evaluate this approach with more serum samples and analyze biochemical changes with more studies during the cancer process
Rian Ka Praja, 2022 [32]	Discriminates the elderly with a low and high percentage of pathogenic CD4+ T Cells	Exploratory study	22 samples	Unscrambler software version 10.4 and SPSS Statistics for Windows version 17.0	Scanning range from 4000 to 650 cm^−1^ with 64 scans and at a 4 cm^−1^ spectral resolution. Analysis parameters: spectral band area ratios and second derivative spectra, calculated by the Savitzky–Golay algorithm	ML-assisted ATR-FTIR spectroscopy can be effectively applied for investigating an immunological alteration in immunosenescence	Required improving the ML methods to assess the real diagnostic performance
Zozan Guleken, 2022 [33]	Characterization of COVID-19-infected pregnant women	Clinical study	37 pregnant women	JASCO Spectra Manager version 2 and OPUS software	Scanning range from 4000 to 400 cm^−1^ with 128 scans (evaluation of the serum spectral bands). Analysis parameter: partial least-squares analysis of the FTIR data, identifying the peak regions and wavenumbers	The obtained FTIR spectra showed significant differences between COVID-19 women with severe and light symptoms	Small sample size
Youssef El Khoury, 2022 [34]	Neuromyelitis optica spectrum	Clinical study	60 samples	OPUS 7.2 software	Scanning range in the mid-IR region, from 3000 to 700 cm^−1^ (higher intensity bands in the 1185 to 950 cm^−1^ region). Analysis parameter: amplitudes of the average spectra and second derivative spectra	The random forest classification ML algorithm distinguished the FTIR second derivatives of serum samples from relapsing–remitting multiple sclerosis (RRMS), NMOSD, and peripheral neuropathy (NEUR) patients	The small number of instances for each serostatus was insufficient to identify specific patterns in the infrared spectra of the sera
Shanshan Guo, 2022 [35]	Tract cancers	Proof-of-concept	210 patients	OPUS 7.2 software	Scanning range in the mid-IR region, from 4000 to 400 cm^−1^, with 32 scans (main bands identified in the 1700 to 1400 cm^−1^ region). Analysis parameters: normalized absorbance amplitude and second derivative spectrum, calculated using the Savitzky–Golay algorithm	There is potential to apply FTIR-based ML diagnosis to aid clinical decision-making as a triage method for digestive tract cancers (DTCs) and for early cancer screening	Reduced number of blood samples and patients from different classes
Riccardo Di Santo, 2022 [36]	Circulating extracellular vesicles	Pilot study	20 patients	OPUS 8.5 SP1 software	Scanning range from 4000 to 650 cm^−1^ with 54 scans and at a 2 cm^−1^ spectral resolution (main spectral peak at 1740 cm^−1^). Analysis parameter: normalized absorbance amplitude	FTIR spectroscopy appears to be extremely promising as it can provide a label-free comprehensive molecular fingerprint of EVs through the analysis of specific mid-IR absorption bands	Small sample size
Zozan Guleken, 2022 [37]	Miscarriage	Clinical study	60 patients	MATLAB Simulink environment (MathWorks, Natick, MA, USA)	Scanning range in the mid-IR region, from 4000 to 600 cm^−1^, at a 4 cm^−1^ spectral resolution. Analysis parameter: average value of absorbance at the 1762, 2874, and 2930 cm^−1^ peaks, after normalization and the average was smoothed by the Savitzky–Golay method	The prediction of lipid profile abnormalities in maternal serum can significantly improve the patient pathway	Required more experimental work
Hongjun Chen, 2022 [38]	Esophageal cancer	Pilot exploratory study	136 patients	MATLAB (Release 2016a)	Scanning range in the mid-IR region, from 4000 to 400 cm^−1^, at a 4 cm^−1^ spectral resolution and 1 s integration time (main intensity peak at 1656 cm^−1^). Analysis parameter: mean and second derivative of the spectra	Principal component-linear discriminant analysis (PC-LDA) with FTIR differentiated esophageal squamous cell carcinoma (ESCC) from normal groups	Required the participation of a large number of patients from different clinical centers
Fengye Chen, 2022 [39]	Ovarian cancer	Clinical study	174 patients	Python 3.7 version	Scanning range from 3200 to 400 cm^−1^ with 2976 spectral channels (main peaks identified in the 1700 cm^−1^ to 800 cm^−1^ region). Analysis parameters: peak positions, peak width, and total spectral intensity (of the normalized spectra)	Raman spectroscopy revealed the importance of certain biomolecules in the identification of the targeted health status of patients	Required more experimental work
Alessandra Koehler, 2022 [40]	Paracoccidioidomycosis	Clinical study	224 patients	Pirouette 4.5 and OriginPro70	Scanning range from4000 to 650 cm^−1^ with 8 scans and at a 4 cm^−1^ spectral resolution (main bands at 1652 cm^−1^ and 1543 cm^−1^). Analysis parameter: second derivative, based on the Savitzky–Golay filter, applied on the average spectrum (normalized)	Able to correctly diagnose paracoccidioidomycosis sera with different clinical forms and degrees of severity	Required more experimental work
Dilek Yonar, 2022 [41]	Malignant pleural mesothelioma	Experimental study	112 patients	Unscrambler X 10.3	Scanning range from 4000 to 650 cm^−1^ with 100 scans and at a 4 cm^−1^ spectral resolution. Analysis parameters: band positions and integrated area ratios between the bands of the average spectra (normalized with respect to the amide I band)	The potential value of the FTIR spectroscopy technique to serve as a screening tool targeting those with a history of asbestos exposure	Required more experimental work
Xiangxiang Zheng, 2022 [42]	Echinococcosis	Clinical study	86 patients	OPUS 7.2 software	Scanning range from 4000 to 600 cm^−1^ with 32 scans and at a 4 cm^−1^ spectral resolution. Raman spectral range from 3100 to 600 cm^−1^. Analysis parameters: intensity differences at some spectral peaks of the normalized spectra using the Savitzky–Golay filter	Explored the use of serum vibrational ATR-FTIR spectroscopy technology combined with an ML algorithm to distinguish echinococcosis patients from healthy individuals	Needed larger clinical cohorts to verify the standardization of serum spectral collection
Nikolas Mateus Pereira de Souza, 2023 [43]	Dyslipidemia types	Clinical study	74 patients	Not reported	Scanning range from 4000 to 650 cm^−1^ with 4 scan pulses and at a 4 cm^−1^ spectral resolution. Analysis parameters: average spectra (normalized between 0 and 1) and the first Savitzky–Golay derivative	ATR-FTIR spectroscopy was associated with chemometric modeling as a plausible applicant for screening different types of dyslipidemia	Extensive studies should be conducted to verify the real applicability in clinical analysis laboratories or medical clinics
Nikolas Mateus Pereira de Souza, 2023 [44]	Breast cancer	Exploratory study	74 patients	ChemoStat V.2	Scanning range from 4000 to 650 cm^−1^ with 4 scan pulses and at a 4 cm^−1^ spectral resolution (main peaks identified in the 1118 to 1052 cm^−1^ region). Analysis parameter: absorbance amplitude in each wavenumber	This methodology plausibly allowed for the screening of the molecular subtypes of breast cancer and consequently improved the prognosis	The centrifugation step to obtain blood plasma and the sample drying step eliminated interference from the water bands
Jingrui Dou, 2023 [45]	Gallbladder cancer	Clinical study	256 patients	OPUS 7.2 software	Scanning range from 4000 to 650 cm^−1^ with 32 scans and at a 4 cm^−1^ spectral resolution (main peaks identified in the 1710 to 1475 cm^−1^ and 1354 to 980 cm^−1^ regions). Analysis parameter: second derivative spectra based on the Savitzky–Golay algorithm	FTIR methods with RBF-SVM methods have the potential to become a serological analysis technique for the early screening of gallbladder cancer	Required more experimental work
Hongyong Leng, 2023 [46]	Cancer prediction	Experimental study	164 blood samples	MATLAB (Release 2016a)	Scanning range from4000 to 600 cm^−1^ with 6 scans and at an 8 cm^−1^ spectral resolution. Raman spectral range from 2000 to 500 cm^−1^. Analysis parameter: amplitude of the average spectrum (normalized between the minimum and maximum values of the data)	The spectral fusion technology effectively improved the accuracy of diagnosis in the complex clinical diagnosis environment	The diagnosis of complex diseases required further experiments to promote practical clinical applications in the future
Luis Ramalhete, 2023 [47]	Discrimination of T and B lymphocytes	Clinical study	18 patients	OPUS 7.2 software	Scanning range from 4000 to 400 cm^−1^ with a 2 cm^−1^ spectral resolution. Analysis parameter: second derivative spectra based on the Savitzky–Golay filter	The ability to economically identify lymphocyte activation in a simple procedure	Future work on the classification models and understanding of the detected spectral difference
Nikolas Mateus Pereira de Souza, 2023 [48]	Metabolic syndrome	Translational clinical study	74 patients	Jasp 0.14.1	Scanning range from 4000 to 650 cm^−1^ with 4 scans and at a 4 cm^−1^ spectral resolution (main peaks identified in the 1800 to 900 cm^−1^ region). Analysis parameter: mean (with standard deviation) of the total spectrum, normalized between 0 and 1	ATR-FTIR spectroscopy with OPLS-DA modeling was powerful enough to discriminate with 100% accuracy individuals with metabolic syndrome from control subjects	Required more experimental work
Salmann Ali, 2023 [49]	Hepatitis C virus	Clinical study	105 patients	Unscrambler X 10.5	Scanning range from 4000 to 400 cm^−1^ with a 4 cm^−1^ spectral resolution (main peaks identified in the 4000 to 502 cm^−1^ region). Analysis parameter: spectra absorbance amplitude	ATR-FTIR spectroscopy in conjugation with multivariate data classification tools holds the potential not only to effectively diagnose hepatitis C virus infections but also the non-cirrhotic/cirrhotic status of patients	Needed for multiple screening tests, used conventionally in order to determine the cirrhotic or non-cirrhotic status of infected individuals

**Table 2:** Methods, metrics, and validation in the selected studies.

Reference	Machine Learning Method *	Metrics to Evaluate ML (Accuracy, Sensitivity, and Specificity, Respectively) (%)	Internal Validation
Emmanuel P. Mwanga, 2019 [13]	NB, XGB, MLP, LR, KNN, RF, and SVM	92; 92.8; 91.7	Cross-validation and external validation
Philip Heraud, 2019 [14]	PLS-DA and SVM	n.a.; 92; 97	Cross-validation
Suat Toraman, 2019 [15]	MLPNN and SVM	95; 93.33; 97.50	Cross-validation (four, five, and ten folds)
Adam H. Agbaria, 2020 [16]	SD, XGB, Gaussian Naïve Bayes, SFSD, Random Forest, and SVM	n.a.; 92; 86	Five-fold validation
Ahmad Salman, 2020 [17]	RF and SVM	88; n.a.; n.a.	Cross-validation
Zozan Guleken, 2020 [18]	PCA, LDA, SVM, and AYC	100; 100; 100	Not conducted
Elke Korb, 2020 [19]	PCA, PDS, TPR, PPV, and CNN	93.9; n.a; 96.5	Ten-fold cross-validation
Adam H. Agbaria, 2020 [20]	SFSD, PCC, and SVM	95; 94; 90	Cross-validation
Feilong Yue, 2020 [7]	MLP, LSTM, and CNN	90; 90; 90	Ten-fold cross-validation
Hyunku Shin, 2020 [21]	PCA, PCA-LDA, CNN, and SVM	88; 84; 85	Five-fold cross-validation
Zozan Guleken, 2020 [22]	PCA, PLS, XGB, HCA, C5.0 decision tree algorithm, KNN, DNN, RF, and SVM	90; n.a.; n.a.	Not conducted
Hugh J. Byrne, 2020 [23]	PLSR	96.67; 90; 100	Root mean square error of cross-validation and cross-validation
Ashton G. Theakstone, 2021 [24]	PCA, HCA, SIMCA, PLS-DA, LDA, and RF	n.a.; n.a.; n.a.	Cross-validation
Fangfang Chen, 2021 [25]	PSO-SVM, PCA, and BP	87.07; n.a.; 90.96	Not conducted
Omar Anwar Elkadi, 2021 [26]	PLS-DA	84.4; n.a.; n.a.	Cross-validation
Rock Christian Tomas, 2022 [27]	PPV, NPV, NB, SVM, LDA, DT, LR, CNN, FNN, and RF	96; n.a; n.a.	Ten-fold cross-validation
Abicumaran Uthamacumaran, 2022 [28]	AdaBoost, decision trees, random forest classifier, and SVM	90; 90; 90	Five-fold cross-validation
Zozan Guleken, 2022 [29]	PLS, SD, DNN, and RF	81.07; n.a.; n.a.	Leave-one-out cross-validation
O.K. Gasymov, 2022 [30]	PCA, sPLS-DA, RF, and SVM	80; n.a.; 90	Cross-validation
Yu Du, 2022 [31]	BPNN and SVM	n.a.; 100; 100	Ten-fold cross-validation
Rian Ka Praja, 2022 [32]	PCA, PLS-DA, NN, RF, and SVM	93.9; n.a.; n.a.	Not conducted
Zozan Guleken, 2022 [33]	PCA, MCC, XGB, DL, kNN + L25, and SVM	100; n.a.; n.a.	Leave-one-out cross-validation
Youssef ElKhoury, 2022 [34]	RF	100; 100; 100	Two-fold cross-validation
Shanshan Guo, 2022 [35]	2D-SD-IR, PLS-DA, MVLR, PCA, RF, KNN, DT, and SVM	95.3; n.a; n.a	Ten-fold cross-validation
Riccardo Di Santo, 2022 [36]	PCA-LDA	n.a.; 95; 95	Leave-one-out cross-validation
Zozan Guleken, 2022 [37]	PCA, PCA-LDA, LDA, and SVM	97.06; 100; 92.85	K-fold cross-validation
Hongjun Chen, 2022 [38]	EA, PC-LDA, PLS-DA, DT, KNN, and SVM	99.26; 98.53; 100	Ten-fold cross-validation
Fengye Chen, 2022 [39]	BPNN	n.a.; 81.0; 97.3	Five-fold cross- validation
Alessandra Koehler, 2022 [40]	PCA and OPLS-DA	100; 100; 100	Leave-one-out cross-validation and error of cross-validation
Dilek Yonar, 2022 [41]	PCA, SVM, and LDA	88.9; n.a.; n.a.	Full cross-validation
Xiangxiang Zheng, 2022 [42]	SVM	97.4; 100; 94.5	Leave-one-out cross-validation, hold-out validation, and five-fold cross-validation
Nikolas Mateus Pereira de Souza, 2023 [43]	PCA and OPLS-DA	100; n.a.; n.a.	Leave-one-out cross-validation
Nikolas Mateus Pereira de Souza, 2023 [44]	PCA and OPLS-DA	100; n.a.; n.a.	Leave-one-out cross-validation and cross-validation
Jingrui Dou, 2023 [45]	PCA, LDA, PCA-LDA, SVM, and RBF-SVM	91.62; 95.83; 86.41	Leave-one-out cross-validation
Hongyong Leng, 2023 [46]	CNN-LSTM, MFCNN, and SVM	97; n.a.; n.a.	Five-fold cross-validation
Luis Ramalhete, 2023 [47]	t-SNE, k-NN, and SVM	99; n.a.; n.a.	Cross-validation
Nikolas Mateus Pereira de Souza, 2023 [48]	OPLS-DA and PLS-DA	n.a.; 100; 100	Leave-one-out cross-validation, root mean square error of cross-validation, and cross-validation
Salmann Ali, 2023 [49]	PCA-LDA, PCA-QDA, and SVM	100; 100; 100	Leave one out cross-validation, cross-validation, and external validation

* Acronyms and abbreviations (alphabetical order): BPNN—backpropagation in neural network; CNN—convolutional neural networks; DL—deep learning; DNN—deep neural networks; DT—decision tree; EA—evolutionary algorithm; FNN—feedforward neural networks; HCA—hierarchical cluster analysis; KNN—K-nearest neighbors classifier; LDA—linear discriminant analysis; LR—logistic regression; LSTM—long short-term memory networks; MCC—Matthews correlation coefficient; MFCNN—multi-frame convolutional neural network; MLP—multilayer perceptron; MLPNN—multi-layer perceptron neural network; NB—Naïve Bayes; NPV—negative predictive value; PCA—principal component analysis; PCC—probability of correct classification; PDS—piecewise direct standardization; PLS—partial least-squares regression; PPV—positive predictive value; RF—random forest; SFSD—soft fixed-complexity sphere decoder; SIMCA—soft independent modelling by class analogy; SVM—support vector machine; TNR—true negative rate; TPR—true positive rate; XBG—extreme gradient boosting; t-SNE—t-distributed stochastic neighbor embedding; OPLS-DA—orthogonal projections to latent structures discriminant analysis; PCA-LDA—principal component analysis and linear discriminant analysis; PCA-QDA—principal component analysis and quantitative descriptive analysis; RBF-SVM—radial basis function kernel and support vector machines; PLS-DA—partial least-squares-discriminant analysis.

## Data Availability

Not applicable.
